# Research on a Real-Time Estimation Method of Vehicle Sideslip Angle Based on EKF

**DOI:** 10.3390/s22093386

**Published:** 2022-04-28

**Authors:** Wen Sun, Zhenyuan Wang, Junnian Wang, Xiangyu Wang, Lili Liu

**Affiliations:** 1 College of Automotive Engineering, Changzhou Institute of Technology, Changzhou 213001, China; sunw@czu.cn (W.S.); 19230427@czu.cn (Z.W.); 19220321@czu.cn (X.W.); liull@czu.cn (L.L.); 2 School of Mechanical and Aerospace Engineering, Queen’s University Belfast, Belfast BT7 1NN, UK; 3 State Key Laboratory of Automotive Simulation and Control, Jilin University, Changchun 130022, China

**Keywords:** extended Kalman filter (EKF), vehicle sideslip angle, least squares method, dynamical model, root mean square error (RMSE)

## Abstract

In this article, a real-time vehicle sideslip angle state observer is proposed, which is based on the EKF algorithm. Firstly, based on a 2-DOF dynamical model and the tire lateral force model, the vehicle sideslip angle state observer model with a self-adapted truncation procedure is established by combining the EKF and the least squares methods. The calculation of the Jacobi matrix in the time domain is transformed into a calculation in the frequency domain. A self-adapted update noise estimation method and an initial value setting strategy are proposed as well. Finally, a hardware-in-the-loop simulation is carried out by Matlab/Simulink-CarSim-dSPACE, and the real-time reliability of the estimation method is verified and analyzed by RMSE.

## 1. Introduction

As an integral part of human daily life, safety is continuously optimized and improved as a premier consideration. Stability control is the main issue to ensure driving handling safety. The reduction in handling stability caused by understeering or oversteering always results in safety hazards and traffic accidents [1]. Therefore, a limiting condition of a vehicle with instant and effective regulation plays a central role in the guarantee of driving safety. The general solution throughout the automotive industry is to regulate vehicles at the right time. In order to achieve these goals, the effective parameter of vehicle state is observed, in which the sideslip angle is a key parameter affecting the vehicle state seriously [2], and is also one of the basic parameters within many control methods [3].

The angle between the longitudinal direction and the direction of motion is called the sideslip angle. The sideslip angle can be measured directly by optical or GPS sensors, but sensors cannot be applied to vehicles due to their high cost and low accuracy. At present, most scholars usually use the method of estimation to obtain the sideslip angle. The sideslip angle is obtained indirectly by measuring the lateral acceleration, the yaw rate, and some other basic parameters of the vehicle during the process of moving. But the uncertainty of modeling in the system, the nonlinear system, and external environmental disturbance factors will result in an increase in the observation error of vehicle sideslip angle. When the lateral acceleration of the vehicle is about to reach the limit value, the steady-state yaw moment of the vehicle corresponding to the same increment of the front wheel angle will decrease with the increase in vehicle sideslip angle, which makes it very difficult to control the yaw moment through the steering wheel [4]. The sideslip angle can be used to judge whether the vehicle is limited during movement. The correction of the target yaw rate can be solved by the estimation result of the vehicle sideslip angle.

In recent years the estimation of the sideslip angle has been studied by scholars using limited measurement data. Most of the methods are based on the kinematics model or the dynamics model [5,6]. The kinematics model analyzes the vehicle motion pattern from geometry, including changes in spatial position or velocity over time. When the vehicle is driven at low speed on good road conditions, dynamics issues are generally not considered. The sideslip angle observer is highly reliable [7]. The dynamical model converts the power output by the engine into the traction force of the vehicle through the force and reaction between the tires and the road. The vehicle’s power system is composed of an engine, which can provide power [8]. The kinematics model reflects vehicle position, vel ocity, and acceleration versus time. The application of the kinematics model in the process of vehicle trajectory planning can make the planned trajectory more realistic and meet the kinematic geometric constraints in the driving process. The dynamical model focuses on the relationship between forces and motion. Dynamical models are generally used to analyze ride comfort and operational stability. The dynamical model is mainly used to study the forces on tires and on their associated components. A relatively accurate mechanical model can be provided by analyzing the mechanical properties of the vehicle under most working conditions and limiting conditions. Most current methods are based on the dynamical model, which is adapted to estimate the sideslip angle of the traditional vehicle or distributed front-drive vehicles.

The main methods for estimating the vehicle sideslip angle are particle filter [9,10], sliding film algorithms [11], direct integration [12], fuzzy logic and neural networks [13], non-linear observers, and Kalman filter algorithms [14,15]. Daniel Chindamo proposed a vehicle kinematic-model-based sideslip angle estimation method by fusing the information from an inertial measurement unit and global navigation satellite system (GNSS), with aligning the heading from the GNSS [16]. Boada transformed the nonlinear dynamical model into an adaptive fuzzy neural network system, and obtained the initial sideslip angle using an adaptive fuzzy neural network. By using UKF to filter the noise, the estimated mean square deviation is minimized to obtain the vehicle sideslip angle [17]. Wenbo Chu proposed a joint observation method based on unscented particle filtering of parameters for distributed electric vehicles. The longitudinal velocity and sideslip angle were observed by a nonlinear dynamic tire model fusing information drive torque and inertial sensors [18]. Xiaoyu Li used a kinetic approach and kinematic geometry methods to estimate the vehicle sideslip angle. The kinematic geometry model between the front and rear wheel slip angle is fused in EKF observers [19]. Jing Li applied the cubature Kalman filter by predefining multiple estimated models and statistical properties of the noise using a model transfer matrix to fuse the multi-model outputs, and maintain the sub-model outputs with small tracking errors. However, the method proposed in this paper has limited applicability for cases where the noise signal is known by default [20]. Xinjiang Jin designed an interactive multi-model vehicle state observer formed by the UKF estimator based on linear and non-linear tire models, and compared it with the IMM-EKF algorithm. The IMM-EKF observer would yield more accurate observations and better robustness under limiting conditions. Since the method proposed in this paper needs to calculate the Jacobi matrix, a large number of calculations will affect the timeliness of the system. [21]. Hong Ding proposed a UKF algorithm-based vehicle sideslip angle estimator. Compared with the UKF algorithm under the same conditions, this estimator achieves higher accuracy and takes less time than the primitive EKF algorithm [22]. Qiu Xia proposed a method for estimating the sideslip angle based on redundant information fusion. The characteristics and applicability of the dynamical model estimator and kinematic model estimator are analyzed. The adaptive weight dynamic adjustment is proposed to fully combine the advantages of the dynamical model observer and kinematic model observer [23]. Gong Wang constructed a dual adaptive volumetric Kalman filter algorithm for the estimation of tire lateral force and vehicle sideslip angle based on the NACKF algorithm for the coupling characteristics of different subsystems of the vehicle, forming closed-loop feedback [24]. Tommaso Novi proposed an integrated artificial neural network and unscented Kalman filter observer using only inertial unit measurements, which can work as a standalone sensor. Direct integration with integral damping and integral reset values allow estimation of the longitudinal velocity using the kinematics model [25]. Table 1 summarizes the current state of research.

Based on the literature review, vehicle state observers can be divided into several categories: The vehicle state is observed by a GPS signal combined with a simple algorithm. The method proposed in this paper has simple steps to obtain the vehicle sideslip angle. However, the method proposed in this paper requires the consumption of high-cost hardware equipment to obtain relatively accurate data in comparison to other methods. If low-cost equipment is used, the proposed method will result in low accuracy. Based on the particle filter and neural network of the vehicle state observer, highly accurate data can be obtained under a lot of working conditions. However, the attenuation problem and the weight need to be considered. As a result of the requirement for resampling and extensive calculation of each wheel, it cannot be applied to vehicles. In using the general Kalman filter, the vehicle state observer has the advantages of light computational requirements and high calculation speeds, except for poor relative accuracy. Other scholars have already proposed various algorithms, such as EKF, UKF, and other optimization algorithms, which can effectively improve observer accuracy. But these optimization algorithms require heavy calculating operations. Compared with other methods, we describe the innovation of this article in detail. In summary, currently available vehicle condition observers can provide relatively accurate estimates under more complex working conditions, and their external anti-interference has also been improved. However, the model structure is usually complex and cannot be effectively applied to the vehicle; thus, online control is not possible. Compared with the above articles, the novelties of this article are the following:

- We propose a calculation method that transitions from the time domain to the frequency domain. The problems of slow calculation speeds and long operation times of the Jacobian matrix are effectively solved.
- We propose an adaptive noise updating method. Aiming at the accuracy of the observer, good results have been achieved.
- We propose a method to update the initial state value, which reduced a lot of iterations. The observer has better timeliness.

The framework of the article is structured as follows. We propose a simpler and faster estimation method. Firstly, the vehicle sideslip angle state observer model with adaptive correction process is established, which is based on the two degrees of freedom dynamical model and tire lateral force model, combined with EKF and the least squares method. Then, a calculation method that transitions from the time domain to the frequency domain of the Jacobi matrix is used. The method proposed in this paper better improves the speed of the algorithm. We use the adaptive noise updating method and the method of updating the initial state. These two methods can greatly improve the estimation speed and accuracy of the observer. Finally, the estimation method proposed in this article is validated based on the Matlab/Simulink-CarSim-dSPACE hardware-in-the-loop simulation platform. In order to verify the superiority of this observer, we calculate the root mean square of the actual vehicle sideslip angle, the estimated value of the primitive observer, and the estimated value of the method proposed in this article. The experimental data and results have fully analyzed the method and algorithm adopted in this article and have achieved good results in the experiment.

## 2. 2-DOF Dynamical Model

**Figure 1:**
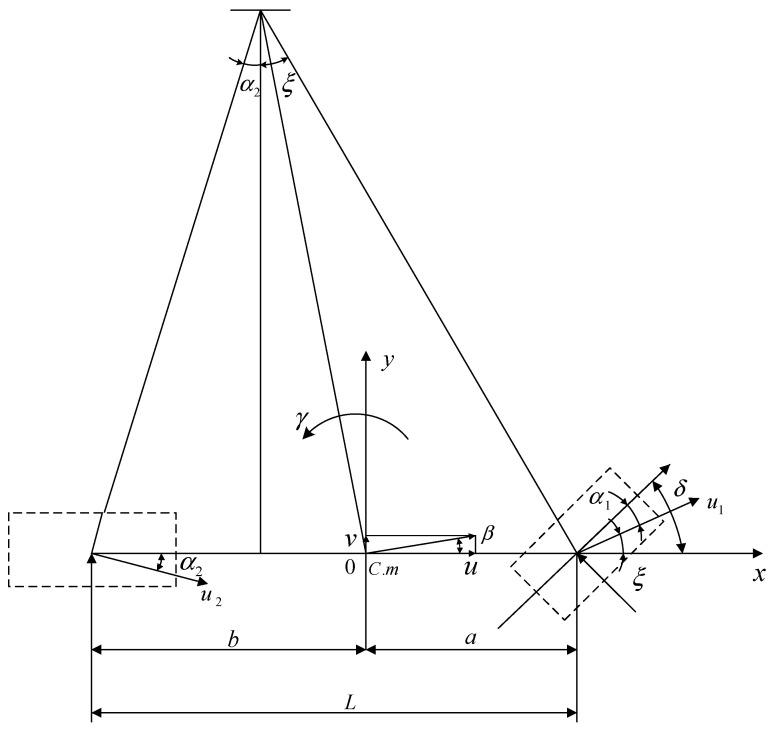
2-DOF dynamical model. Where $\alpha_{1}$, $\alpha_{2}$ are front and rear wheel sideslip angles, respectively; $u_{1}$, $u_{2}$ are speeds at the midpoint of automotive front and rear axles, respectively; ξ is the angle between $u_{1}$ and the *x*-axis; L is the wheelbase; u is the forward speed; v is the lateral speed; $k_{1}$, $k_{2}$ are the cornering stiffnesses of the front and rear wheels, respectively; a, b are the distances from the vehicle center of gravity to the front and rear axles; δ is the front steering angle; m is the vehicle mass; $I_{z}$ is the equivalent yaw moment of inertia; β is the vehicle sideslip angle; γ is the yaw rate; C.m is the vehicle centroid.

The research focus is the lateral motion of the vehicle stability control. 2-DOF describes the basic motion forms of the vehicle, such as lateral and yaw motion. It reflects the tire cornering characteristics of the moving vehicle, and it can also describe the vehicle motion state in most working conditions accurately. At the same time, we consider the complexity and timeliness of the observer, thus we choose 2-DOF as the reference model. The centroid of the model is coincident with the coordinate origin, and the mechanical properties of each tire are considered in the linear range. The model has the same left and right front wheel angles, and ignores the effect of longitudinal rolling resistance. The 2-DOF dynamical model is shown in Figure 1.

The equations of the lateral and yaw motions of the vehicle model are expressed as follows:

(1) $$m(\dot{v}+u\gamma )=k_{1}\delta -(k_{1}+k_{2})\beta -\frac{ak_{1}-bk_{2}}{u}\gamma$$

(2) $$I_{z}\dot{\gamma }=ak_{1}\delta -(ak_{1}-bk_{2})\beta -\frac{a^{2}k_{1}+b^{2}k_{2}}{u}\gamma$$

The vehicle status parameters are listed in the following Table 2.

Kalman filtering algorithm estimates the optimal data for a linear system from the collected observations. The optimal estimate process can be considered as data processing disturbed by noise. The vehicle state is a nonlinear change process when the 2-DOF model is combined with the Kalman filter algorithm. Therefore, this article adopts the algorithm based on EKF for the vehicle sideslip angle.

### Extended Kalman Filtering Algorithm

The extended Kalman filter (EKF) is an extended form of the Kalman filter in the nonlinear case. It is an efficient recursive filter that applies to nonlinear systems.

The equation of a nonlinear discrete system state can be expressed as follows.

(3) $$x_{k+1}=f(x_{k},u_{k})+Q_{k}$$

The system measurement equation can be expressed as follows.

(4) $$y_{k}=g(x_{k},u_{k})+R_{k}$$

where, f(x,u) and g(x,u) are nonlinear functions; $Q_{k}$, $R_{k}$ are the system noise and measurement noise, respectively.

The observer is a model that expands the nonlinear functions f(x,u) and g(x,u) around $x_{k}$, using Taylor series to obtain an approximately linear system. The EKF is divided into the prediction phase and the correction phase. The current moment’s system state value is used as input to estimate the forecast estimate for the next moment in the forecast phase. The observations and prediction estimates are fused to obtain the optimal estimate in the correction phase. The steps of general EKF are shown as follows:

The prediction phase can be expressed as follows:

(5) $$x_{k}^{\prime }=f(x_{k-1})$$

(6) $$y_{k}^{\prime }=F_{k-1}y_{k-1}F_{k-1}{}^{T}+Q$$

The correction phase can be expressed as follows:

(7) $$S_{k}^{\prime }={\left(H_{k}y_{k}^{\prime }H_{k}{}^{T}+R\right)}^{-1}$$

(8) $$K_{k}^{\prime }=y_{k}^{\prime }H_{k}{}^{T}S_{k}^{\prime }$$

(9) $$x_{k}=x_{k}^{\prime }+K_{k}^{\prime }(y_{k}-g(x_{k}^{\prime }))$$

(10) $$y_{k}=(I-K_{k}^{\prime }H_{k})y_{k}^{\prime }$$

where F and H are the partial derivatives of the functions f, h, respectively, at $x=x_{k}$.

(11) $${F_{k}=\frac{\partial f}{\partial x}|}_{x=x_{k}^{\prime }},\;{H_{k}=\frac{\partial g}{\partial x}|}_{x=x_{k}^{\prime }}$$

Solving the Jacobi matrix requires a lot of calculation. In this article, we propose a transformation from calculating the Jacobian matrix in the time domain to calculating the Jacobian matrix in the frequency domain. The Fourier transform was used to transform the function in the time domain into the frequency domain for calculation.

The function f(x) will perform the Fourier transform as follows:

(12) $$F(\omega )=\int_{-\infty }^{+\infty }f(t)e^{-i\omega t}dt$$

Inversing the Fourier transform Equation (12) yields the following:

(13) $$f(t)=\frac{1}{2\pi }\int_{-\infty }^{+\infty }F(\omega )e^{i\omega t}d\omega$$

where, i, j and ω are constants; t is the variable. The function to the frequency domain was Fourier transformed, and the Jacobian matrix was calculated in the frequency domain. The obtained Jacobian matrix is transformed into the time domain via inverse Fourier transform. When F[x(t)]=X(jω), the integral and differential equations in the frequency domain are shown as follows:

(14) $$F^{-1}\left[\frac{dX(j\omega )}{d\omega }\right]=(-jt)x(t)$$

(15) $$F^{-1}\left[\int_{-\infty }^{\infty }X(j\omega )d\omega \right]=\frac{x(t)}{-jt}+x(0)\pi \delta (t)$$

In the EKF algorithm, computation of the Jacobi matrix in each iteration takes a lot of time, hence the general EKF observer cannot have good real-time performance. In this article, we propose a real-time estimation method of vehicle sideslip angle based on EKF, which transforms from calculating the Jacobian matrix in the time domain to calculating the Jacobian matrix in the frequency domain. It has a simpler algorithm that significantly reduces operations, and has better real-time performance.

## 3. Vehicle Sideslip Angle Estimator

**Figure 2:**
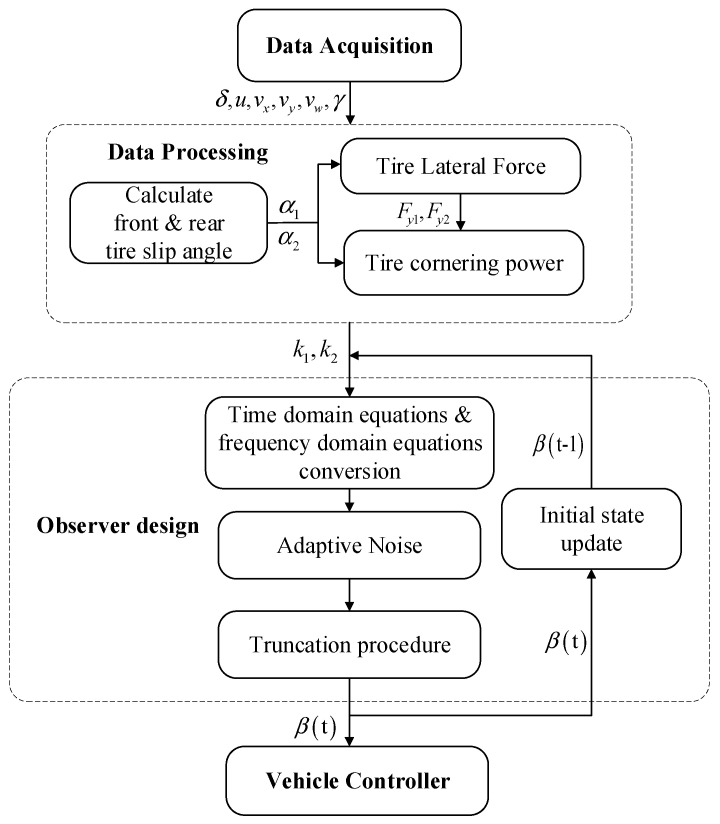
Algorithm flow chart.

In this article, in order to achieve real-time observation, the sideslip angle estimator can be expressed as shown in Figure 2. The signals of front-wheel angle, speed, and wheel angle are collected in data acquisition. Data processing will deal with the collected signals. The tire lateral force and tire cornering stiffness are obtained according to the tire model. The observer estimates the sideslip angle, combining the tire cornering stiffness, front-wheel angle, and longitudinal speed. The initial parameters of the observer will be set to the last observed value for faster iterations. Finally, the sideslip angle of the observer is fed to the vehicle controller for the relevant state regulation.

### 3.1. Model State Space Description

The equations of state and measurement for a nonlinear system can be expressed as follows.

(16) $$\dot{x}(t)=f(x(t),u(t))+Q(t)$$

(17) y(t)=g(x(t),u(t))+R(t)

where, x(t)=[β;γ]; $y(t)=[a_{y};\gamma ]$, β, and γ are the same as described previously; $a_{y}$ is the lateral acceleration; u(t)=δ(t), where δ(t) is the input of the front steering angle with time.

The 2-DOF equations are incorporated into Equations (16) and (17) to obtain the following:

(18) $$\left[\begin{array}{c} \dot{\beta }\\ \dot{\gamma } \end{array}\right]=\left[\begin{array}{cc} \frac{k_{1}+k_{2}}{mv} & \left(\frac{ak_{1}-bk_{2}}{mv^{2}}-1\right)\\ \frac{ak_{1}-bk_{2}}{I_{z}} & \left(\frac{a^{2}k_{1}+b^{2}k_{2}}{I_{z}v}\right) \end{array}\right]\left[\begin{array}{c} \beta \\ \gamma \end{array}\right]+\left[\begin{array}{c} -\frac{k_{1}}{mv}\\ -\frac{ak_{1}}{I_{z}} \end{array}\right]\delta$$

(19) $$\left[\begin{array}{c} a_{y}\\ \gamma \end{array}\right]=\left[\begin{array}{cc} -\frac{k_{1}+k_{2}}{m} & \frac{ak_{1}-bk_{2}}{mv}\\ 0 & 1 \end{array}\right]\left[\begin{array}{c} \beta \\ \gamma \end{array}\right]+\left[\begin{array}{c} \frac{k_{1}}{m}\\ 0 \end{array}\right]\delta$$

The initial state parameter of the system is $x_{0}=[0;0]$.

This article uses the semi-empirical magic tire equation to calculate the lateral force and the front and rear tire cornering stiffnesses. The tire lateral force is calculated by the equation as follows:

(20) y=Dsin{Carctan{Bx−E[Bx−arctan(Bx)]}}

where, x is the tire slip angle α or slip rate κ; B is the stiffness factor; C is the form factor; D is the peak factor, and E is the curvature factor. The tire slip angle α and slip rate κ can be expressed as follows:

(21) $$\alpha =\arctan(\frac{v_{y}}{v_{x}})$$

(22) $$\kappa =\frac{v_{x}-v_{r}}{v_{x}}$$

The front and rear wheels slip angles are expressed in the coordinate system as follows:

(23) $$\alpha_{1}=\beta +\frac{a\gamma }{u}-\delta_{f}$$

(24) $$\alpha_{2}=\beta -\frac{b\gamma }{u}$$

where $v_{x}$ is the longitudinal velocity of the wheel center; $v_{y}$ is the lateral velocity of the wheel; $v_{r}=\omega R$, where ω is the angular velocity of wheel rotation, and R is the wheel rolling radius.

### 3.2. Noise Update

Noise will impact the whole observation system. Gaussian white noise is usually selected by observers as the noise of the whole system. Gaussian white noise’s power spectral density obeys a uniform distribution; Gaussian white noise is not correlated and is independent. However, in the process of estimation, the white noise may possess greater errors as a result of constant iterations. Gaussian white noise is represented below as WGN.

In this article, an adaptive noise cycle system is used to analyze and update the noise for each iteration. The noise update equations are represented as follows:

(25) $$q_{k}=(1-\frac{1}{k})q_{k-1}+\frac{1}{k}[x_{k}-f(x_{k-1})]$$

(26) $$r_{k}=(1-\frac{1}{k})r_{k-1}+\frac{1}{k}[y_{k}-g(x_{k}^{\prime })]$$

(27) $$\lambda_{k}=y_{k}-g(x_{k}^{\prime })-r_{k}$$

(28) $$Q_{k}=(1-\frac{1}{k})Q_{k-1}+\frac{1}{k}[K_{k}\lambda \lambda^{T}K_{k}{}^{T}+y_{k}-F_{k-1}y_{k-1}F_{k-1}{}^{T}]$$

(29) $$R_{k}=(1-\frac{1}{k})R_{k-1}+\frac{1}{k}[\lambda \lambda^{T}-H_{k}y_{k}^{\prime }H_{k}{}^{T}]$$

where q, r, λ are defined parameters, which are used to solve for Q and R.

**Figure 3:**
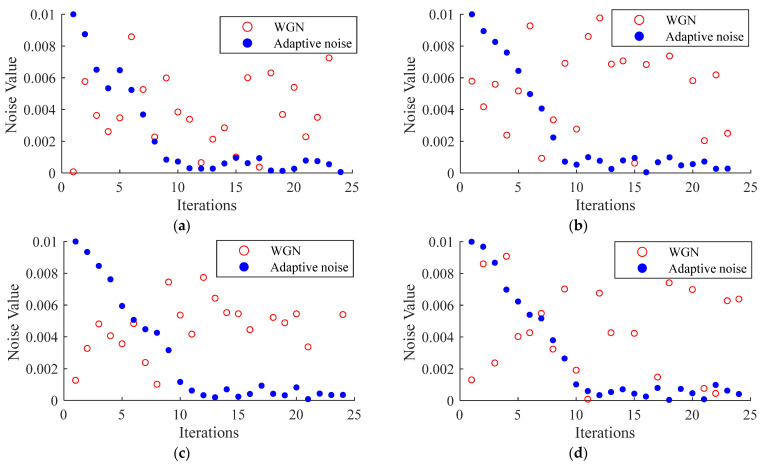
WGN and adaptive noise contrast chart. (**a**) Noise comparison in the first iteration; (**b**) noise comparison in the second iteration; (**c**) noise comparison in the third iteration; (**d**) noise comparison in the fourth iteration.

In this article, the effect of adaptive noise on the system is verified by adding Gaussian white noise and adaptive noise to certain sets of data in the iterations of the observation process, which can be shown in Figure 3.

The iterative noise change during the observer processing is compared with Gaussian white noise. In Figure 3, the mean value of Gaussian white noise is 0 and the variance is 0.01 in the process of targeting a certain iteration, which does not have a correlation in the iteration and is randomly distributed. For the adaptive noise, the initial value is 0.01. The noise changes with the updated iteration of the system, and the noise stabilizes at around 0.001 in 10 iterations. Primitive Gaussian white noise is uncorrelated and independent, which cannot follow the correlation of the system during the iterative process. This reduces the resistance of the system to external disturbances. In this article, we propose an adaptive noise updating strategy to better fit the external environment. With continuous iterations, it is obvious that the noise gradually fits the system to reach the final relatively stable value, thus the accuracy of the observer can be further improved.

### 3.3. Adaptive Truncation Strategy

The EKF algorithm requires several iterative processes. A truncation strategy based on the least squares method is proposed in this article. When the residual sum of squares of all observations in the function ε is minimized to obtain the optimal parameter solution θ, the initial least squares equation can be expressed as follows:

(30) $$\min\varepsilon =\sum_{i=1}^{n}{\left(f(x_{i})-y_{i}\right)}^{2}$$

Its higher-order function can be expressed as follows:

(31) $$\min\varepsilon ={\left|\theta \vec{X}-\vec{Y}\right|}^{2}$$

The optimal parameter θ can be expressed as the following:

(32) $$\theta ={\left(X^{T}X\right)}^{-1}X^{T}Y$$

where X is the observed value and Y is the theoretical measurement. When θ is within the error band of 5% for three consecutive iterations, the observed value can be considered to be close to the true value, and the iteration is finished.

### 3.4. Design of Initial Values of Parameters

Considering that the Kalman filter is gradually approximating the true value in iterations, more iterations are needed to be used to approximate the true value when the initial state is too different from the true value. In this article, the initial state of each iteration is set to be the estimation result of the previous moment in order to solve the observed timeliness problem. The initial state equation is expressed as follows:

(33) $$x_{0}(t)=x_{k}^{\prime }(t-1)$$

**Figure 4:**
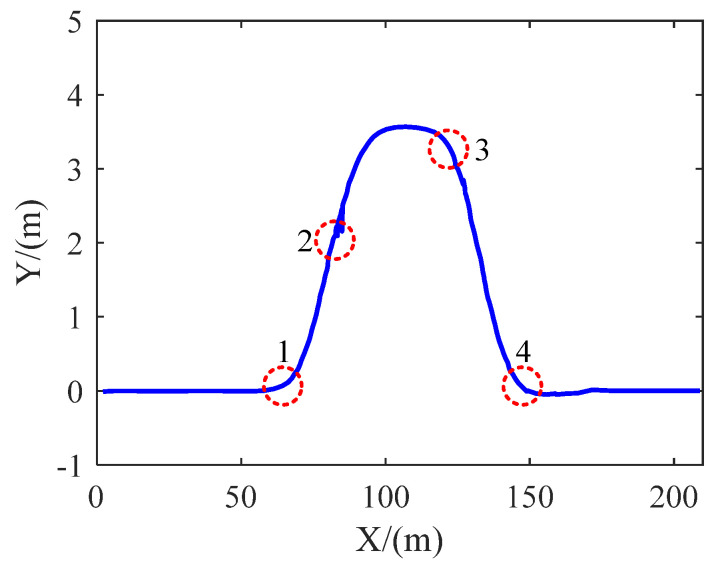
Routing and point selection in double lane change. (The red dotted circles are randomly selected four points).

In order to verify the timeliness of the observer, this article uses offline co-simulation of Simulink-CarSim. The working condition is the double shift line working condition, and the simulation time is 15 s. The number of iterations of the program after different modifications is compared with the actual running time, and four points are randomly selected from the real motion trajectory as reference points, which are shown in Figure 4.

**Figure 5:**
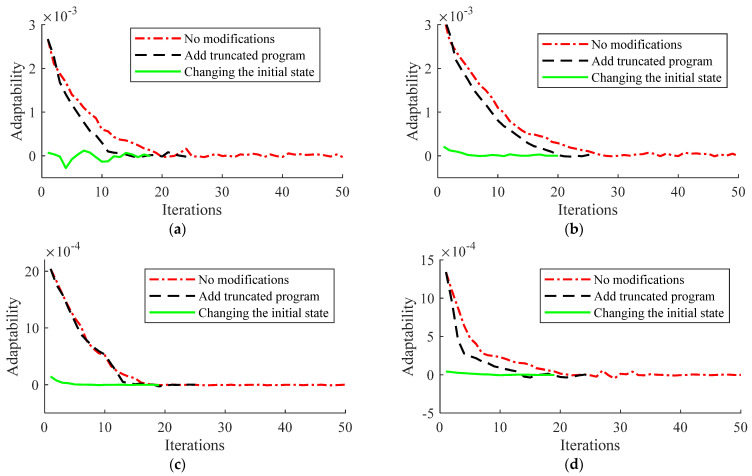
Adaptation curve comparison chart. (**a**) The adaptability of the three methods is at the first point; (**b**) the adaptability of the three methods is at the second point; (**c**) the adaptability of the third method is at the first point; (**d**) the adaptability of the three methods is at the fourth point.

Figure 5 shows a comparison of the adaptation curves for three cases of reference points: an observer with no program changes, adding a truncated program, and changing the initial state. The equation can be expressed as the following:

(34) $$s(x)=x_{k}^{\prime }-y_{k}$$

where s(x) denotes the point’s value in fitness, $x_{k}$ denotes the predicted value of the point at each iteration, and $y_{k}$ denotes the theoretical actual value of the point. The curves obtained by the three methods are compared and analyzed. No modifications mean that the observed values are filtered, but the results are not processed. Add truncated program means that the observer can selectively truncate the program according to the final estimated value. Changing the initial state means that the initial value of the system is changed to be the observation result of the previous time.

As shown in Figure 5a, the curves are compared and analyzed. In the case of no additions, the initial value default is 0, which differs greatly from the true value and requires several iterations to approach the true value. Program termination is entirely unrelated to the approach of the true value, with a default iteration of up to 50 generations excluding the truncation strategy. Iterating is up to about 23 generations. The observed value is close to the true value and tends to be stable by the red dotted line. The truncation strategy is designed to be shown as the black dashed line, in which the predetermined error range is reached at 23 iterations, and the procedure is truncated. It effectively reduces the number of operations and saves time. When the initial state is close to the true value, as shown by the green solid line, fewer iterations are experienced to reach the specified error range, further reducing the number of iterations.

The number of iterations and time for the four points in the three cases are also compared and analyzed. The process of Kalman filtering is usually set to 50 iterations to ensure that the operation is completed. The number of iterations and time for the three methods is shown in the Table 3. 

When the initial state does not change, the initial state of the observer defaults to 0. Using Figure 5a as an example, it can be analyzed that the observer takes 50 iterations to finish when no additions are made. The observations are close to the true value when it reaches 25 iterations. With the addition of the truncation procedure, it is clear that the speed of iteration increases. However, when it reaches 23 iterations, the procedure is truncated to avoid redundant operations. After changing the initial state, it is evident that the iteration speed increases again, and iterations are truncated at 18. Thus, the speed of operations has been greatly improved.

The comparative analysis of the four sets of adaptation curves in Figure 5 shows that when the initial state is changed, the number of iterations after changing the initial state is less than that with no change, which can quickly approach the true value. This means that the number of iterations can be effectively reduced by changing the initial state, and the computing time is significantly shortened, which can provide better real-time performance.

Firstly, compared with the procedure without any modifications, the number of iterations is nearly doubled with the addition of the truncation procedure, and the computation time is reduced to 45% of the original time. When the initial state is modified to the previously estimated value, the number of iterations is reduced to 36% of the original number, and the time is reduced to 32% of the original time. Compared with the results of the truncation program, the number of iterations is reduced by 22%, and the time is reduced by 28%.

Secondly, compared with the procedure without any modifications, the number of iterations is nearly doubled with the addition of the truncation procedure, and the computation time is reduced to 49% of the original time. When the initial state is modified to the previously estimated value, the number of iterations is reduced to 42% of the original number, and the time is reduced to 34% of the original time. Compared with the results of the truncation program, the number of iterations is reduced by 16%, and the time is reduced by 29%.

The third point is that compared with the procedure without any modifications, the number of iterations is nearly doubled with the addition of the truncation procedure, and the computation time is reduced to 44% of the original time. When the initial state is modified to the previously estimated value, the number of iterations is reduced to 40% of the original number, and the time is reduced to 32% of the original time. Compared with the results of the truncation program, the number of iterations is reduced by 17%, and the calculation time is reduced by 23%.

The fourth point is that compared with the procedure without any modifications, the number of iterations is nearly doubled with the addition of the truncation procedure, and the computation time is reduced to 44% of the original time. When the initial state is modified to the previously estimated value, the number of iterations is reduced to 36% of the original number, and the time is reduced to 30% of the original time. Compared with the results after adding the truncation process, the number of iterations is reduced by 22%, and the calculation time is reduced by 31%.

To sum up, compared with the original scheme, the number of iterations can be reduced by nearly half, and the time can be reduced by nearly 45% by adding a truncation procedure. On the basis of adding the truncation program, we changed the initial conditions of the program. The number of iterations can be reduced to about 40% of the original program, and the time can be shortened by nearly 33%. The results show that adding a truncation program and changing the initial state can effectively reduce the amount of calculation, and shorten the operation time on the premise of ensuring accuracy.

## 4. Estimation and Simulation of the Sideslip Angle

**Figure 6:**
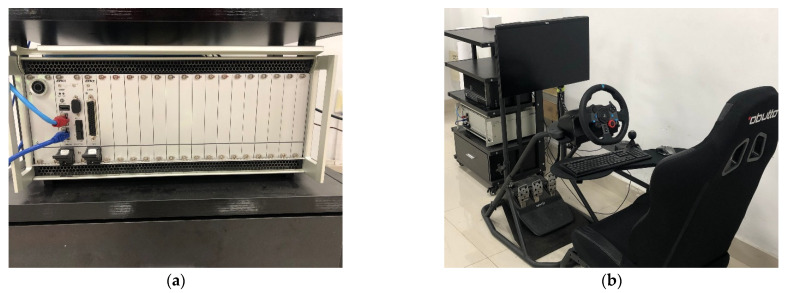
Simulation equipment. (**a**) dSPACE SCALEXIO; (**b**) driving simulator.

We adopted MATLAB/Simulink-CarSim-dSPACE for hardware in the loop simulation. A co-simulation program with CarSim was created in Simulink. The steering wheel angle, accelerator pedal opening, and brake pedal opening were entered by the real driver. HIL simulation was carried out at the same time. HIL simulation is the simulation system that directly puts part of system hardware that needs to be simulated into the simulation loop, which can not only make up for many of the shortcomings in purely digital simulation and increase the confidence level of the overall model, but this also can greatly reduce the programming effort. The simulation device model used in this article is the dSPACE SCALEXIO LabBox, as shown in Figure 6.

Connect the dSPACE simulation device with the driving simulator and add the data measured by the real driver to the mathematical model, to conduct a simulation.

**Figure 7:**
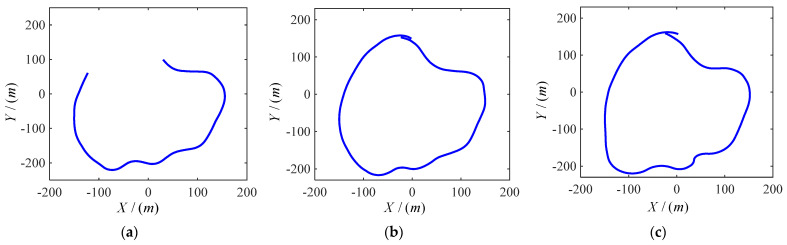
Vehicle real travel trajectories. (**a**) The track at moderate and low speed; (**b**) track at moderate and high speed; (**c**) track at high speed.

We choose a road model. The model is controlled by the driver and simulated at different speeds. By using CarSim to output the parameter required by sideslip angle observation and conducting real-time simulation analysis, we can obtain three different kinds of real trajectories, which are shown in Figure 7.

**Figure 8:**
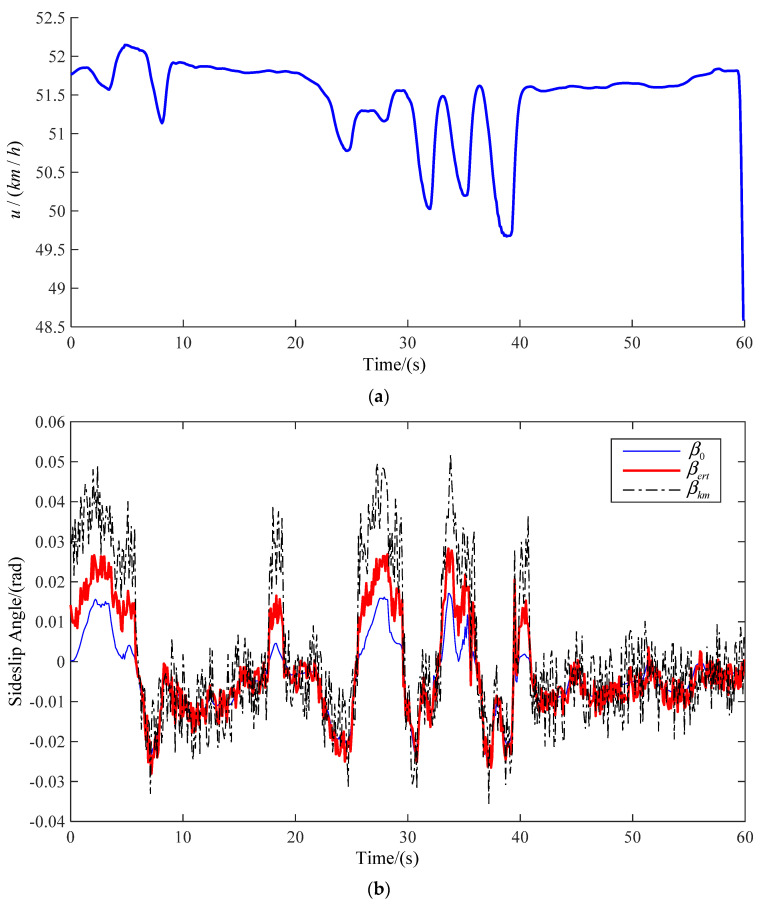
Medium and low speed (**a**) real-time speed; (**b**) estimation of sideslip angle.

**Figure 9:**
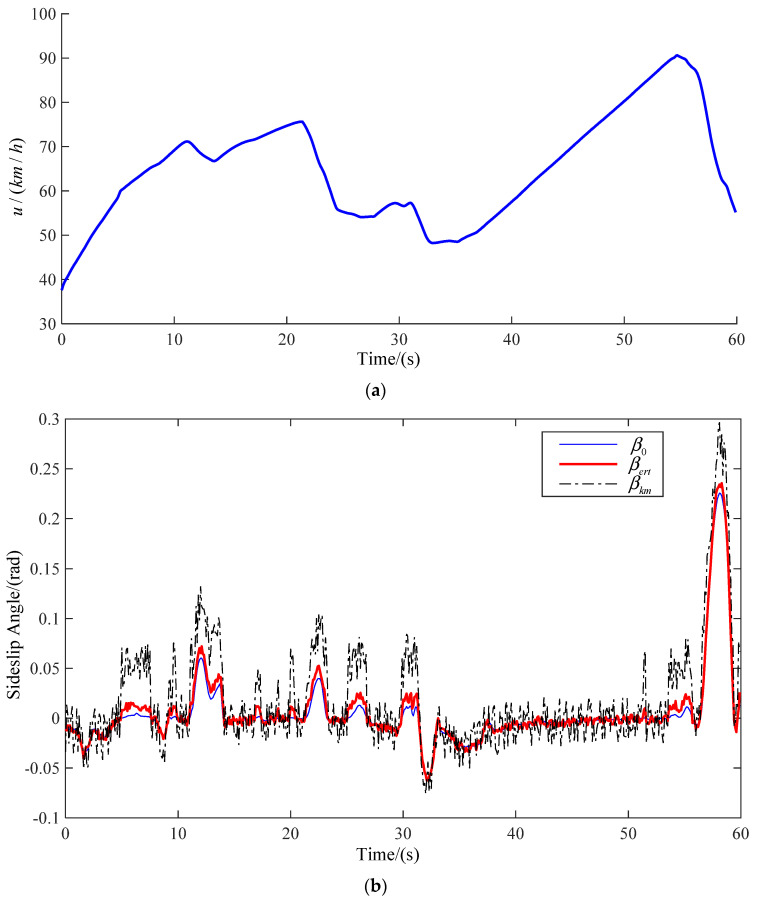
Medium and high speed (**a**) real-time speed; (**b**) estimation of sideslip angle.

**Figure 10:**
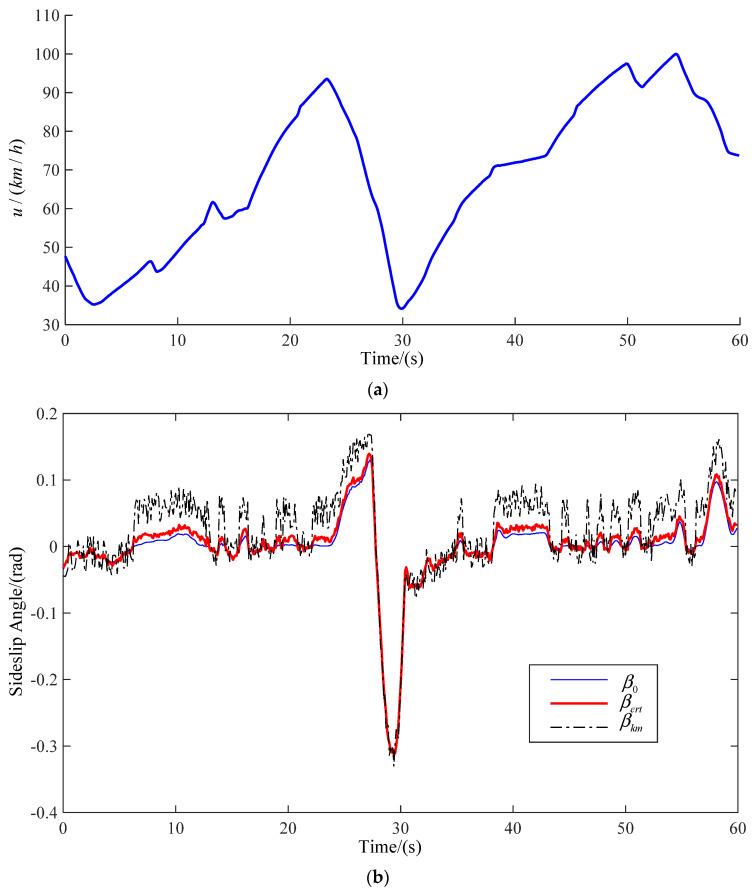
High speed (**a**) real-time speed; (**b**) estimation of sideslip angle.

The road model selected in this paper includes many simple working conditions, and can be used to verify and analyze the method proposed in this paper comprehensively. The real-time speed and vehicle sideslip angle at three speeds are observed. The observation results are shown in Figure 8, Figure 9 and Figure 10, respectively, corresponding to Figure 7a–c. The blue line shows the actual vehicle sideslip angle curve, which is denoted as $\beta_{0}$; the black dashed line shows the vehicle sideslip angle curve observed by the primitive Kalman filter observer, which is denoted as $\beta_{\mathrm{km}}$; the red solid line shows the sideslip angle curve observed by the EKF-based vehicle sideslip angle estimation strategy proposed in this article, which is denoted as $\beta_{\mathrm{ert}}$.

In order to show the influence of vehicle speed change on centroid sideslip angle better, the ordinates of the three vehicle speed change curves are not the same. The ordinate of Figure 7a is 48.5–52.5. The ordinate of Figure 7b is 30–100. The ordinate of Figure 7c is 30–110. However, the true value of vehicle centroid sideslip angle cannot be obtained directly. In order to verify the feasibility of the method, we believe that the sideslip angle of the CarSim output is the real value. The driver controls the vehicle to drive on the same road at different speeds. In Figure 8, when the vehicle is driven at medium and low speed, the vehicle has low requirements for speed when turning, hence the fluctuation of sideslip angle is small. In Figure 9 and Figure 10, when the vehicle is driven at medium and high speed, proper deceleration is required to ensure stability of the vehicle when turning. When the speed is high, the vehicle sideslip angle centroid is large, but the estimated value is relatively accurate. This is because the Kalman filter is a motion-based estimation method. At high speed, the vehicle sideslip angle changes greatly, and the estimated value is relatively more accurate.

The actual vehicle sideslip angle $\beta_{0}$, the observations of a common Kalman filter observer $\beta_{km}$, and the observations of an EKF-based real-time observer $\beta_{ert}$, are compared and analyzed in this article. The vehicle speed will decrease when turning, and the vehicle sideslip angle will change. The common Kalman filter observer yields good observation results only for a linear system, but the actual vehicle state parameter is a nonlinear system. Using a common Kalman filter observer to estimate the sideslip angle when the vehicle turns will produce a large error. At the same time, due to the essence of the Kalman filter, when the vehicle speed changes greatly and the sideslip angle changes greatly, the estimated data are closer to real values. The EKF-based real-time observer is an observer for nonlinear systems, thus it has higher observational accuracy. The results show that the observation results based on EKF-based real-time observers are closer to real values.

In order to better illustrate the advantages of the method proposed in this article, we compare the root mean square error (RMSE) of three groups of data. RMSE is sensitive to the maximum/minimum errors in a series of measurements, hence RMSE can be used to well reflect the measuring accuracy [26,27]. RMSE reflects the offset degree between the measured data and true values. Smaller RMSE promotes data accuracy. The calculation equation of RSME can be expressed as shown below:

(35) $$M=\sqrt{\frac{\sum_{i=1}^{n}{\left(\beta_{i}-\beta_{0}\right)}^{2}}{n}}$$

where M is root-mean-square error; n is several data, $\beta_{0}$ is the true value of the vehicle sideslip angle, and $\beta_{i}$ is the observed value. The calculation results are shown in Table 4.

By comparing the estimated results of the primitive Kalman filter observer with the results of the EKF-based adaptive observer, we can see that there is a large deviation between the estimated results of the primitive Kalman filter observer and the actual vehicle sideslip angle. The estimation results of the method presented in this article are closer to the true values, and only show small errors when cornering. In Figure 8, Figure 9 and Figure 10, $\beta_{ert}$ is closer to the real curve. As shown in Table 4, at medium and low speed, the RMSE value of the method proposed by us is 0.0055, and the RMSE of primitive Kalman filter observer is 0.0142. Compared with the primitive Kalman filter observer, the accuracy of the method proposed by us is improved by 60%. At medium and high speed, the RMSE value of the method proposed by us is 0.0057, and the RMSE of primitive Kalman filter observer is 0.0314. Compared with the primitive Kalman filter observer, the accuracy of the method proposed by us is improved by 81%. At high speed, the RMSE value of the method proposed by us is 0.0073, and the RMSE of primitive Kalman filter observer is 0.0386. Compared with the primitive Kalman filter observer, the accuracy of the method proposed by us is improved by 81%. In conclusion, the proposed method in this article is better than the traditional observation method.

## 5. Conclusions

1. Through the theoretical analysis of a 2-DOF dynamical model combined with the EKF algorithm, this article explained the importance of vehicle sideslip angle observer in vehicle control.
2. The vehicle sideslip angle state observer was built based on EKF. Aimed at the complicated process of calculating the Jacobian matrix in the EKF algorithm, the calculation in the time domain was transformed into the frequency domain. In this article, the partial derivative calculated in the time domain was converted to the frequency domain, which made its calculation simpler.
3. An adaptive noise updating method was proposed in this article. The adaptive noise in an iteration process was compared with Gaussian white noise. The comparative analysis showed that the adaptive noise updating method effectively reduced the interference of noise to the system and made the estimation results more accurate.
4. By further analyzing the EKF-based observer, an adaptive truncation system based on the least squares method and initial state update system was proposed in this article. Through the co-simulation by Simulink-CarSim, four random points under double line change were selected as the reference points to compare the operation speed of the method proposed in this paper and other methods. Simulation analysis showed that the number of iterations used by the method proposed in this article was 36% of original methods, and the number of times was 32% of original methods. Experiments showed that the method proposed in this article has better timeliness.
5. Finally, real-time simulations were carried out by Matlab/Simulink-CarSim-dSPACE. The change curves of vehicle sideslip angle under different driving conditions were output through real-time control by the driver. The root mean square under the three working conditions was calculated. The curve and root mean square values effectively showed the superiority of the method proposed in this paper.

In this article, the algorithm, noise, and initial state were optimized to improve the observer accuracy, reduce the number of operations, and shorten the operation time. In this article, vehicle sideslip angle was observed, but there was no effective control over the vehicle. Aiming at the problem of vehicle stability control, we will continue to study the control algorithm when the observation results reach the stability limit.

## Figures and Tables

**Table 1:** Current status of research.

Methods Involved	Targeted Questions	Method Features
GPS signal-based method	An estimation method that addresses the limited accuracy and high price of GPS.	Estimate higher accuracy state values by adding simple algorithms using only GPS signals.
Fuzzy neural network-based approach	Solving the problem of insufficient accuracy of primitive observers in the nonlinear region of the car.	The nonlinear vehicle system is transformed into an adaptive fuzzy neural network system, and the transverse eccentricity of the center of mass is estimated by other algorithms.
Kalman filter-based estimation method	The linear problem of the Kalman filter is transformed into a nonlinear problem of UKF or EKF, and the vehicle sideslip angle is observed.	Generally, the dynamical model is added to the observer model, and the observation method that the observed value is close to the real value is continuously iterated by algorithms such as UKF or EKF.
Estimation method based on tire model	Simulation of real vehicle forces by tire model and observation of vehicle sideslip angle according to dynamical model.	Based on a variety of semi-empirical tire models, the vehicle forces are estimated from simple vehicle parameters to calculate the vehicle’s sideslip angle. Additional algorithms are usually added after the force analysis to make the estimation more accurate.
Estimation method based on synovial control	Slip film control algorithm-based observer design for vehicle sideslip angle observation.	The saturation function is introduced as the switching function when observing vehicle sideslip angle based on the slip film observer, which reduces the jitter phenomenon caused by the sign function.
Particle filtering-based estimation method	Vehicle dynamics model combined with particle filtering algorithm for vehicle state observation.	The main improvements are simplifying the complex iterative process and proposing a method to effectively solve the degeneracy linearity while allowing a more accurate selection of action points.

**Table 2:** Vehicle status parameters.

Parameters	Values
Vehicle mass, *m* (kg)	1270
Rated load, *F_N_* (N)	3000
Tire free radius, *R* (m)	0.317
Distance from the front axle to gravity center, *a* (m)	1.015
Distance from the rear axle to gravity center, *b* (m)	1.895
Equivalent yaw moment of inertia, *I_z_* (kg/N^2^)	1536.7

**Table 3:** The number of iterations and time for the three methods.

	Program Modification	No Modifications	Add Truncated Program	Changing the Initial State
Time and Number of Iterations				
First point		3.233/50	1.428/23	1.033/18
Second point		3.567/50	1.768/25	1.267/21
Third point		3.297/50	1.467/24	1.134/20
Fourth point		3.557/50	1.596/25	1.099/20

**Table 4:** The root mean square error of three speeds.

Working Condition	$\beta_{ert}$	$\beta_{km}$
Medium and low speed	0.0055	0.0142
Medium and high speed	0.0057	0.0314
High speed	0.0073	0.0386

## Data Availability

Not applicable.
